# Chronic kidney disease, heart failure and neprilysin inhibition

**DOI:** 10.1093/ndt/gfz058

**Published:** 2019-04-26

**Authors:** Richard Haynes, Doreen Zhu, Parminder K Judge, William G Herrington, Philip A Kalra, Colin Baigent

**Affiliations:** 1 Medical Research Council Population Health Research Unit, University of Oxford, Headington, Oxford, UK; 2 Clinical Trial Service Unit and Epidemiological Studies Unit, Headington, Oxford, UK; 3 Department of Renal Medicine, Salford Royal NHS Foundation Trust, Stott Lane, Salford, UK

**Keywords:** blood pressure, cardiovascular, CKD, heart failure, renin-angiotensin system

## Abstract

Patients with chronic kidney disease are at increased risk of cardiovascular disease and this often manifests clinically like heart failure. Conversely, patients with heart failure frequently have reduced kidney function. The links between the kidneys and cardiovascular system are being elucidated, with blood pressure being a key risk factor. Patients with heart failure have benefitted from many trials which have now established a strong evidence based on which to base management. However, patients with advanced kidney disease have often been excluded from these trials. Nevertheless, there is little evidence that the benefits of such treatments are modified by the presence or absence of kidney disease, but more direct evidence among patients with advanced kidney disease is required. Neprilysin inhibition is the most recent treatment to be shown to improve outcomes among patients with heart failure. The UK HARP-III trial assessed whether neprilysin inhibition improved kidney function in the short- to medium-term and its effects on cardiovascular biomarkers. Although no effect (compared to irbesartan control) was found on kidney function, allocation to neprilysin inhibition (sacubitril/valsartan) did reduce cardiac biomarkers more than irbesartan, suggesting that this treatment might improve cardiovascular outcomes in this population. Larger clinical outcomes trials are needed to test this hypothesis.

## CKD AND STRUCTURAL HEART DISEASE ARE CLOSELY ASSOCIATED

Chronic kidney disease (CKD) and heart failure (HF) frequently coexist and both are associated with high morbidity and mortality [1, 2]. Numerous studies have shown that there is an inverse association between kidney function and cardiovascular risk [3, 4]. Structural heart disease, which may manifest clinically as HF, is a leading cause of cardiovascular disease in CKD patients and its prevalence increases with declining kidney function [2, 5]. A cross-sectional echocardiographic observational study reported an increasing prevalence of left ventricular hypertrophy (LVH) with decreasing estimated glomerular filtration rate (eGFR) (from 32% among patients with eGFR ≥60 mL/min/1.73 m^2^ to 75% among patients with eGFR <30 mL/min/1.73 m^2^) [6, 7]. Studies using cardiac magnetic resonance imaging with gadolinium enhancement have found that diffuse late gadolinium enhancement is associated with the degree of LVH [8] and indicates myocyte disarray and interstitial fibrosis histologically [9]. Although overt systolic dysfunction is not common (affecting only 8% of patients in the above cross-sectional echocardiographic study) and not clearly associated with kidney function [7], more subtle disturbances in ventricular function (such as reduced left ventricular deformation, early myocardial relaxation velocity or reduction in global longitudinal strain that may contribute to diastolic dysfunction) are more common and are present even in the early stages of CKD [10, 11]. These abnormalities provide the anatomical substrate for the excess risk of symptomatic HF, arrhythmia and sudden cardiac death observed among patients with advanced CKD. Conversely, in large HF registries, 20–68% of patients with HF have moderate to severe kidney disease [1]. The presence of CKD is associated with poor prognosis in HF and can be used to stratify the risk of patients with HF [6, 12, 13].

## PATHOPHYSIOLOGY OF HF IN CKD

The pathophysiological relationship between the heart and the kidneys involves many different pathways. CKD may disturb homoeostasis in ways that may be directly damaging to the cardiovascular system [i.e. ‘direct’ risk factors such as high blood pressure (BP) or vascular calcification] or the kidneys and circulation may both be subject to ‘indirect’ risk factors (e.g. diabetes mellitus and smoking). In addition, HF may worsen CKD by decreasing renal perfusion, causing renal venous congestion and activation of the sympathetic nervous system and renin–angiotensin–aldosterone system (RAS, which may in turn cause inflammation and oxidative stress). Treatment for HF in CKD can be divided into two broad types: (i) treatments that intervene on pathophysiological links between CKD and HF to prevent HF and (ii) treatments known to improve prognosis in established HF among people without CKD.

## TREATMENT TO PREVENT HF IN CKD

CKD is commonly associated with high BP, due to salt and water retention, activation of the sympathetic nervous and other neurohormonal systems and accumulation of endogenous vasopressors [14]. Studies of living kidney donors suggest that reducing GFR by 10 mL/min as a consequence of donor nephrectomy leads to a 5 mmHg increase in systolic BP [15]. BP is positively associated with the risk of death from HF [16] and randomized trials have demonstrated that this association is causal [17]. Meta-analysis of all the major BP-lowering trials has shown that a 10 mmHg reduction in systolic BP lowers the risk of HF by 28% [95% confidence interval (CI) 22–33] [18]. Most classes of antihypertensive treatments have similar effects, with the exception of calcium channel blockers (which may have a smaller benefit) and diuretics (which may have a larger benefit) [18]. A subgroup analysis within this meta-analysis (which included 13 trials involving nearly 38 000 participants, of whom 6000 had CKD) suggested that the effect of BP lowering on HF was larger among patients without CKD [relative risk (RR) 0.48 (95% CI 0.38–0.62)] than among patients with CKD [RR 0.95 (95% CI 0.70–1.04); P for interaction <0.001] [18]. Nevertheless, the benefits of lowering BP on other cardiovascular outcomes remain clear even among patients with CKD.

Anaemia is a well-recognized complication of CKD and has been proposed as a direct cause of HF in patients with CKD following observational and non-randomized interventional studies, suggesting that anaemia is associated with LVH and correcting the anaemia reverses the LVH [19, 20]. However, randomized trials have shown that full or partial correction of anaemia with erythropoiesis-stimulating agents (ESAs) does not reduce left ventricular mass nor the risk of HF and may even increase the risk of other cardiovascular outcomes such as stroke [21].

Reducing parathyroid hormone concentrations with calcimimetic therapy might reduce the risk of non-atherosclerotic cardiovascular events (such as HF) among haemodialysis patients [22, 23]. Such treatment also reduces fibroblast growth factor 23 (FGF23; see below). Unfortunately, the randomized data on other interventions that target CKD-specific mechanisms of HF are much less robust. For example, although there is evidence that hyperphosphataemia (i) can cause vascular smooth muscle cells to adopt an osteoblastic phenotype and cause vascular calcification (which in turn increases cardiac afterload) [24] and (ii) is associated with LVH [25], no sufficiently large trials of phosphate reduction have been conducted to elucidate whether these associations are causal. Although FGF23 has been found to induce LVH after direct intracardiac injection in mice [26], the totality of the observational evidence does not suggest that FGF23 is a cause of cardiovascular disease (and no trials of FGF23 reduction in CKD exist) [27].

## TREATMENT TO IMPROVE PROGNOSIS IN ESTABLISHED HF IN THE GENERAL POPULATION

The main objectives of HF therapy in CKD (as well as in non-CKD) patients are to decrease the preload and afterload and to reduce LVH, treat myocardial ischaemia and inhibit neurohumoral hyperactivity, especially the sympathetic nervous system and RAS [28]. However, the optimum treatment of HF in patients with CKD remains unclear, as there is little direct evidence to support any recommendations. Most of the pivotal randomized trials that guide the management of HF define CKD as a baseline eGFR <60 mL/min/1.73 m^2^ but have excluded patients with more advanced stages of CKD (i.e. eGFR <30 mL/min/1.73 m^2^).

Many pharmacological and device treatments are recommended for HF with reduced ejection fraction (HFrEF) [29]. The mainstays of such treatment are angiotensin-converting enzyme inhibitors (ACEis) and β-blockers. The largest trial of ACEis in HFrEF was Studies of Left Ventricular Dysfunction (SOLVD)-Treatment, which compared enalapril 10 mg twice daily with placebo among 2569 patients with HFrEF and demonstrated a 16% (95% CI 5–26) reduction in mortality (primary outcome) [30]. This effect was similar in patients with and without CKD [31]. Similarly, in the four large trials of β-blockers in HFrEF, there was no good evidence that the benefits of β-blocker therapy were modified by baseline kidney function. The results of these trials (and their published effects by baseline kidney function) are summarized in Table 1.

**FIGURE 1:**
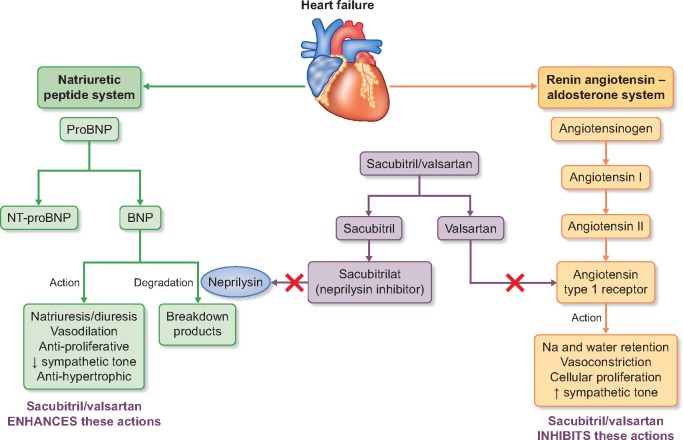
Effects of sacubitril/valsartan on vasoactive peptides.

**Table 1:** Effect of kidney function on the efficacy of established treatments for chronic HFrEF

Trial (ref)	Intervention (sample size)	Main eligibility criteria	Follow-up (years)	Primary outcome	Overall treatment effect (95% CI)	CKD subgroups (eGFR, mL/min/ 1.73 m^2^)	Treatment effect in CKD	P for treatment × CKD interaction
**ACEi**								
SOLVD-TREATMENT [31]	Enalapril versus placebo (*n* = 2569)	LVEF ≤35%; NYHA I–IV; creatinine <177 μmol/L	3.5	All-cause mortality	0.84 (0.74–0.95)	≥60 (*n* = 1466)	0.82 (0.69–0.98)	0.62
						<60 (*n* = 1036)	0.88 (0.73–1.06)	

**β-blocker**								
CIBIS-II [32]	Bisoprolol versus placebo (*n* = 2647)	LVEF ≤35%; NYHA III–IV; creatinine <300 μmol/L	1.3	All-cause mortality	0.66 (0.54–0.81)	<45 (*n* = 450)	0.71 (0.48–1.05)	0.81
						≥45 <60 (*n* = 669)	0.69 (0.46–1.04)	
						≥60 <75 (*n* = 640)	0.53 (0.34–0.82)	
						>75 (*n* = 863)	0.64 (0.42–0.99)	
MERIT-HF [33, 34]	Metoprolol versus placebo (*n* = 3991)	LVEF ≤40%; NYHA II–IV; ‘significant’ kidney disease	1	All-cause mortality	0.66 (0.53–0.81)	<45 (*n* = 493)	0.41 (0.25–0.68)	0.095
						≥45–≤60 (*n* = 976)	0.68 (0.45–1.02)	
						>60 (*n* = 2496)	0.71 (0.54–0.95)	
SENIORS [35, 36]	Nebivolol versus placebo (*n* = 2128)	LVEF <35% or hospitilization for decompensated HF; NYHA II–IV; creatinine <250 μmol/L	1.75	All-cause mortality or CV hospital admission	0.86 (0.74–0.99)	<55.5 (*n* = 704)	0.84 (0.67–1.07)	0.442
						55.5–72.8 (*n* = 704)	0.79 (0.60–1.04)	
						>72.8 (*n* = 704)	0.86 (0.65–1.14)	

**Mineralocorticoid receptor antagonist**								
RALES [37, 38]	Spironolactone versus placebo (*n* = 1663)	LVEF <35%; NYHA III–IV; creatinine ≤221 μmol/L	2	All-cause mortality	0.70 (0.60–0.82)	<60 (*n* = 792)	0.68 (0.56–0.84)	N/A
						≥60 (*n* = 866)	0.71 (0.57–0.90)	
EMPHASIS-HF [39]	Eplerenone versus placebo (*n* = 2737)	LVEF ≤35%; NYHA II; eGFR ≥30 mL/min/1.73 m^2^	1.75	CV death or hospitalization for HF	0.63 (0.54–0.74)	<60 (*n* = 912)	N/A	0.50
						≥60 (*n* = 1821)	N/A	

**Angiotensin receptor neprilysin inhibitor**								
PARADIGM-HF [40]	Sacubitril/valsartan versus enalapril (*n* = 8442)	LVEF ≤40%; NYHA II–IV; eGFR ≥30 mL/min/1.73 m^2^	2.25	CV death or hospitalization for HF	0.80 (0.73–0.87)	<60 (*n* = 3061)	N/A	0.91
						≥60 (*n* = 5338)	N/A	

**ICD**								
MADIT II [41]	Prophylactic ICD versus conventional medical therapy (*n* = 1232)	LVEF ≤30%; NYHA III; eGFR ≥15 mL/min/1.73 m^2^	2.67	All-cause mortality	0.69 (0.51–0.93)	<35 (*n* = 80)	1.09 (0.49–2.43)	0.29
						35–59 (*n* = 387)	0.74 (0.48–1.15)	
						≥60 (*n* = 756)	0.66 (0.43–1.02)	

**CRT**								
CARE-HF [42]	CRT versus conventional medical therapy (*n* = 813)	LVEF ≤35%; NYHA III–IV;	1.5	Death from any cause or unplanned hospitalization for a major CV event	0.63 (0.51–0.77)	<60 (*n* = 369)	0.67 (0.50–0.89)	N/A
						≥60 (*n* = 370)	0.57 (0.40–0.80)	

Data extracted from large trials where subgroup analysis by kidney function is available. NYHA, New York Heart Association; CV, cardiovascular; N/A, not available.

For patients with HFrEF [with a left ventricular ejection fraction (LVEF) <35%] who remain symptomatic after optimization of ACEi and β-blocker therapy, guidelines recommend a mineralcorticoid receptor antagonist (MRA). This recommendation follows two large trials (see Table 1). Again, the effect of treatment on the primary outcome was not modified by baseline kidney function. However, these trials highlight the importance of safety as a consideration in the treatment of patients with CKD. Patients with CKD are at higher risk of hyperkalaemia (due to the reduced ability of their kidneys to excrete potassium), which is associated with an increased risk of hospitalization and death [43]. The trials had stringent monitoring of serum potassium and developed criteria for reducing the dose or stopping the MRA, such that there was no excess death due to hyperkalaemia in the trials. The importance of such monitoring is highlighted by population-based studies, which demonstrate increased rates of hospitalization for hyperkalaemia since the publication of these trials [44]. Device therapies [implantable cardioverter defibrillators (ICDs) and cardiac resynchronization therapy (CRT)] also improve prognosis in selected patients with HFrEF). A meta-analysis of the trials of ICDs has raised the hypothesis that worse kidney function might attenuate the benefit of these devices [45], but this is not the case for CRT devices. Intravenous iron has been shown to improve functional capacity among patients with HFrEF and results of clinical outcomes trials are needed [46]. Indeed, the PIVOTAL trial among haemodialysis patients suggests that intravenous iron may reduce cardiovascular morbidity in this population [47]. This finding may alter the interpretation of the placebo-controlled ESA trials in which participants allocated to placebo received more iron.

However, as noted above, few patients with CKD have HFrEF, whereas structural substrates for diastolic dysfunction are common among patients with CKD. In contrast with HFrEF, no treatment has yet demonstrated convincing benefit (in terms of morbidity and mortality) in patients with HF with moderately reduced EF (HFmrEF: LVEF ≥40–<50%) or HF with preserved EF (HFpEF: LVEF ≥50%). The Treatment of Preserved Cardiac Function Heart Failure with an Aldosterone Antagonist trial tested spironolactone (15–45 mg daily) versus placebo in 3445 patients with LVEF ≥45% and observed a non-significant 11% (95% CI −4–23) reduction in the primary outcome of cardiovascular death, aborted cardiac arrest or hospitalization for HF [37]. There was again no modification of the treatment effect by baseline kidney function. However, *post hoc* analyses have suggested that patients recruited from certain geographic regions had significantly worse adherence to treatment (when measured biochemically), which may have made the overall result a ‘false negative’ [48].

## NEPRILYSIN INHIBITION

Neprilysin [also known as neutral endopeptidase (NEP)] degrades natriuretic and other vasoactive peptides (including bradykinin, substance P, endothelin and angiotensin II) and therefore neprilysin inhibition (NEPi) enhances the activity of the natriuretic peptide system leading to natriuresis, diuresis, BP reduction and inhibition of RAS and the sympathetic nervous system [49]. Isolated NEPi causes reflex activation of the RAS, so development of NEPi has always been combined with ACEi or ARB. The potential of NEPi in HFrEF was suggested in the Omapatrilat versus Enalapril Randomized Trial of Utility in Reducing Events trial, which compared omapatrilat (a combined ACEi and NEPi) to enalapril in 5770 patients with HF and found a non-significant 6% (95% CI −3–14) reduction in the primary outcome of all-cause mortality or hospitalization for HF [50]. However, development of omapatrilat was stopped when the Omapatrilat Cardiovascular Treatment Assessment Versus Enalapril trial (in 25 302 patients with hypertension) found an excess risk of angioedema compared with enalapril (2.17 versus 0.68%; P < 0.005) [51]. This was thought to be due to excessive bradykinin concentrations (as both ACE and NEP degrade bradykinin) and led to the development of a new class of drug called an angiotensin receptor neprilysin inhibitor (ARNI), which combines NEPi with an ARB.

Sacubitril/valsartan is a first-in-class ARNI that is rapidly metabolized after ingestion to the NEPi pro-drug sacubitril and the ARB valsartan. Sacubitril/valsartan reduces BP more than equivalent doses of valsartan alone [52]. The Prospective Comparison of ARNI with ACEi to Determine Impact on Global Mortality and Morbidity in Heart Failure (PARADIGM-HF) trial randomized 8442 participants with HFrEF to treatment with sacubitril/valsartan or enalapril and was terminated earlier than planned based on the recommendation by the Data Monitoring Committee after interim efficacy analysis showed overwhelming evidence of benefit at a median follow-up duration of 27 months. Compared with those assigned to enalapril, participants assigned to sacubitril/valsartan in PARADIGM-HF experienced a 20% (95% CI 13–27) reduction in the primary composite endpoint of cardiovascular death or HF hospitalization. This effect was again similar among participants with and without CKD. Sacubitril/valsartan is now recommended in the European Society of Cardiology guidelines as a replacement for ACEi (or ARB) in patients who have symptomatic HF with a reduced LVEF ≤35% and who remain symptomatic despite maximum-tolerated evidence-based treatment [29, 40].

Sacubitril/valsartan has also been tested among patients with HFpEF. The PARAMOUNT trial compared sacubitril/valsartan with valsartan in 301 patients with change in NT-proBNP as the primary outcome [53]. At 12 weeks, among participants assigned sacubitril/valsartan, NT-proBNP was 23% (95% CI 8–36) lower compared with participants assigned valsartan. The PARAGON-HF trial has recruited 4822 participants with HFpEF to compare sacubitril/valsartan with valsartan and is scheduled to be completed in mid-2019 [54]. The primary outcome is the composite of cardiovascular death and total (first and recurrent) hospitalizations for HF.

In addition to its known benefits in HFrEF (and potential for benefit in HFpEF), NEPi might also have beneficial effects on the kidney. Experiments using 5/6 nephrectomy models suggested that NEPi reduces proteinuria and histological markers of kidney damage more than ACE inhibition alone [55, 56]. In addition, sacubitril/valsartan appeared to slow the deterioration of kidney function in the PARADIGM-HF [57] and PARAMOUNT trials [58]. However, it also modestly increased albuminuria in both trials (although baseline levels were very low in these HF populations) [59].

The UK Heart and Renal Protection (HARP)-III trial was designed to investigate the short- to medium-term effects of sacubitril/valsartan 97/103 mg twice daily versus irbesartan 300 mg once daily on kidney function among patients with established CKD [60]. Patients were eligible for the UK HARP-III trial if either their eGFR was ≥20–<45 mL/min/1.73 m^2^ or their eGFR was ≥45–<60 mL/min/1.73 m^2^ and the urine albumin:creatinine ratio was >20 mg/mmol. Other pre-specified outcomes included albuminuria, BP and cardiac biomarkers. A total of 414 participants were randomized and the average eGFR was 35 mL/min/1.73 m^2^ and median urine albumin:creatinine ratio was 54 mg/mmol. Only 4 and 13% reported HF and coronary heart disease, respectively, at baseline.

The primary outcome of measured GFR at 12 months did not differ between the two groups: the difference in means was −0.1 (standard error 0.7) mL/min/1.73 m^2^ [61]. Albuminuria was not significantly reduced [9% (95% CI −1–18) among those assigned sacubitril/valsartan] despite an additional 5.4/2.1 (both P < 0.001) mmHg reduction in BP. Despite the apparent lack of an effect on short to medium-term kidney function, allocation to sacubitril/valsartan did reduce both NT-proBNP and troponin I compared with allocation to irbesartan. Study average concentations of NT-proBNP and troponin I were 18% (95% CI 11–25) and 16% (95% CI 8–23) lower, respectively.

Although the effects on kidney function are not encouraging, they do not exclude a benefit on long-term progression of CKD (although any effect would not be large). However, the effects on BP and cardiac biomarkers support the hypothesis that sacubitril/valsartan might reduce the risk of cardiovascular events (and in particular those related to HF) among patients with CKD, irrespective of whether they have known cardiac disease. The neutral effects on tolerability and safety outcomes in the UK HARP-III trial would also support further investigation of this hypothesis.

## CONCLUSION

The burden of HF among patients with CKD is considerable and contributes significantly to the excess of cardiovascular morbidity and mortality observed in this growing population. The anatomical substrates of HF develop early in the progression of CKD and strategies to prevent it have not been rigorously tested in the CKD population. Furthermore, trials among patients with known HF have usually excluded patients with moderate or advanced CKD, so the efficacy and—importantly—the safety of these treatments in the CKD population are uncertain. NEPi looks promising as a treatment that could reduce the risk of HF safely among patients with CKD, but clinical outcome trials are required. Newer treatments for HF, such as sodium glucose co-transporter-2 inhibitors, are being tested in large trials in both HF and CKD populations [62–64] and may be the first treatments that have proven efficacy for HF among patients with a wide-spectrum of kidney disease. Nevertheless, further trials of established and future interventions are required that allow doctors to confidently reduce excess risk of cardiovascular disease in CKD.

## FUNDING

RH, PK, WH and CB work at the Clinical Trial Service Unit, University of Oxford which received a grant from Novartis Pharma AG to conduct the UK HARP-III trial. CTSU has also received grants from Merck, Pfizer and Boehringer-Ingelheim for research in kidney disease. CTSU has a staff policy of not accepting honoraria or other payments from the pharmaceutical industry, except for the reimbursement of costs to participate in scientific meetings (www.ctsu.ox.ac.uk).

## CONFLICT OF INTEREST STATEMENT

The UK HARP-III trial was funded by a grant to the University of Oxford from Novartis Pharma. The trial was conducted, analysed and published independently of the funder. See www.ctsu.ox.ac.uk.
